# What We Miss in Patients With Persistent Thrombocytopenia During the Dengue Season

**DOI:** 10.7759/cureus.97273

**Published:** 2025-11-19

**Authors:** Amit Bisht, Ratnil Joshi, Sonu Sama, Akash Rawat

**Affiliations:** 1 Department of General Medicine, Himalayan Institute of Medical Sciences, Swami Rama Himalayan University, Dehradun, IND; 2 Department of Critical Care Medicine, Himalayan Institute of Medical Sciences, Swami Rama Himalayan University, Dehradun, IND

**Keywords:** dengue fever (df), evans syndrome, immune thrombocytopenia (itp), persistent thrombocytopenia, secondary itp

## Abstract

Dengue is an arboviral illness caused by Aedes aegypti and usually presents with fever and thrombocytopenia. This decrease in platelet counts is observed to revert after the seroconversion phase of the infection. On the other hand, immune thrombocytopenia (ITP) is an autoimmune phenomenon with pathological destruction of platelets. It is a diagnosis made only after exclusion of other causes. Evans syndrome is a combination of ITP with autoimmune haemolytic anaemia (AIHA). Here, we present a case series of patients with persistent thrombocytopenia during the dengue season and the aftermath of it at a single tertiary centre in North India, with concluding diagnoses of ITP and AIHA.

## Introduction

Dengue is a viral disease caused by an arbovirus that is transmitted to humans by an infected Aedes aegypti mosquito. Clinical manifestations of dengue represent a spectrum of subclinical syndromes with no or minimal symptoms to severe dengue and life-threatening severe dengue, with thrombocytopenia being a common presentation among patients [1]. Immune thrombocytopenic purpura (ITP) is an autoimmune condition characterised by mucocutaneous bleeding and a low platelet count due to pathogenic antibodies that lead to accelerated platelet destruction and clearance [1,2]. It is classified based on its aetiology (primary and secondary) or based on time elapsed since diagnosis (newly diagnosed, persistent, and chronic ITP). Its occurrence in children usually follows common childhood viral infections and is mostly self-limiting [3]. In adults, viral infections such as human immunodeficiency virus (HIV), hepatitis C virus, varicella-zoster virus, rubella, influenza, Epstein-Barr virus (EBV), and parvovirus B19 have been reported to precede ITP occurrence [4]. This case series depicts the forthcoming and subsequent diagnosis that we made of persistent thrombocytopenia in dengue patients, because usually the thrombocytopenia resolves once the infection subsides, within a period of seven to 10 days. It also brings into the limelight the importance of meticulous and timely diagnosis of ITP after dengue fever, given the fatality of some possible outcomes of the disease, such as massive intracranial bleed, as described in a recent case report [3].

## Case presentation

Case 1

A 20-year-old female patient, primigravida with no known comorbidities, presented to the hospital at 35 weeks of gestation in active labour with severe thrombocytopenia and transaminitis. She had been diagnosed with dengue by a positive enzyme-linked immunosorbent assay (ELISA) test at 34 weeks of gestation. On presentation, her vitals were stable. Abdominal examination revealed epigastric tenderness along with a gravid uterus corresponding to the period of gestation. She was in active labour, and a normal vaginal delivery was done. Complete haemogram showed a haemoglobin (Hb) of 9.43 g/dl (reference: 13-17 g/dl), total leucocyte count of 9.61/mm^3 ^(reference: 4000-10,000/mm^3^), and platelet count of 8400/mm^3^ (reference: 150,000-410,000/mm^3^). Peripheral blood smear showed features of haemolysis with severe thrombocytopenia. Direct Coombs test was strongly positive (3+). On the 10th day of admission, the patient complained of bleeding gums and rashes. An antinuclear antibody (ANA) test was performed, and it was positive for Sjögren's syndrome A (SS-A). There was no history of sicca symptoms, hence Sjögren’s syndrome was ruled out. Relevant investigations, such as scrub ELISA, peripheral smear for malaria, and blood cultures to rule out typhoid fever, were negative, ruling out common infectious causes of thrombocytopenia. Bone marrow examination showed cellular marrow with trilineage haematopoiesis with adequate megakaryocytes. Taking into account the results and by ruling out all causes of thrombocytopenia, the diagnosis of ITP was made. The combination of autoimmune haemolytic anaemia (AIHA) and ITP in a patient confirmed the diagnosis of Evans syndrome. The patient was initiated on intravenous (IV) steroids (dexamethasone 40 mg daily for four days) with gradual improvement in anaemia and thrombocytopenia. The patient was then placed on rituximab maintenance therapy and was discharged. On follow-up, the patient had normal platelet counts (180,000/mm^3^).

Case 2

A 29-year-old female patient, primigravida at 30 weeks and four days of gestation, presented to the hospital with complaints of fever and abdominal pain lasting four days. On examination, the patient had pallor, but no icterus or lymphadenopathy. However, there was mild epigastric tenderness on palpation of the abdomen. Baseline investigations showed a Hb of 11.2 g/dl, thrombocytopenia (10,000/mm^3^), and leukopenia (2970/mm^3^) with mild transaminitis. The patient was diagnosed with dengue fever after non-structural protein 1 (NS1) positivity and was started on IV fluids and other supportive treatment. Gradually, the patient’s general condition improved, but thrombocytopenia persisted in the range of 10,000-20,000/mm^3^ even after a week of being afebrile. The peripheral blood smear depicted markedly reduced platelet counts with preserved platelet morphology. So, a diagnosis of secondary ITP due to a viral infection was suspected. The workup for autoimmune causes was negative. The complete haemogram and peripheral blood smear did not reveal features suggestive of a haematological malignancy. Other common infectious causes, such as malaria, scrub typhus, and typhoid fever, were also ruled out. The patient was counselled for bone marrow examination to rule out haematological malignancy, but refused the same. She was initiated on IV steroids (dexamethasone 40 mg daily for four days) on day seven of the afebrile period, after the diagnosis of secondary ITP was confirmed. The course was continued for four days, and with an increase in platelet count on the third day, her discharge was planned. The patient was discharged on the fifth day on maintenance steroids with a stable platelet count (200,000/mm^3^).

Case 3

**Figure 1:**
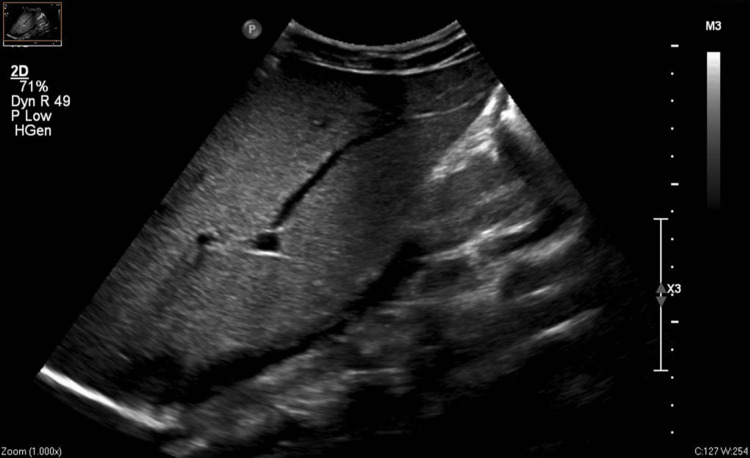
Finding of hepatomegaly on ultrasonography

A 50-year-old female patient, who was a known case of hypertension (not on medication), presented to the hospital with complaints of fever and generalised weakness for five days, rashes all over the body for two days, and gum bleeding for one day. Her physical examination showed no abnormality and her vitals were stable. Basic investigations revealed severe thrombocytopenia (12,000/mm^3^). Workup for dengue NS1/IgM rapid test, scrub ELISA, peripheral smear for malaria, and blood cultures for enteric fever were negative. However, the IgM ELISA for dengue was reported to be positive subsequently. Whole abdomen ultrasonography (USG) showed hepatomegaly with a normal spleen, as shown in Figure 1 below.

The general blood picture showed severe thrombocytopenia with normal platelet morphology. Liver function and kidney function tests were found to be normal. So, a working diagnosis of secondary ITP was made. Bone marrow examination revealed hypercellular marrow with increased megakaryocytes. Hence, the diagnosis of ITP, which had been exacerbated by a viral infection, was confirmed. The patient was initiated on IV steroids (dexamethasone 40 mg daily for four days), and the platelet count improved gradually from 12,000/mm^3^ to 118,200/mm^3^. The patient was successfully discharged on day seven with a platelet count of 129,000/mm^3^.

Case 4

A 44-year-old female patient, known case of breast carcinoma, and status post chemoradiotherapy, with a history of dengue fever two months ago, presented to the hospital with complaints of gum bleeding, black-coloured stools, and persistent thrombocytopenia for the same duration. She had been diagnosed with dengue two months back by a positive ELISA test. Haematological investigations revealed anaemia (Hb=9.4 g/dl) with severe thrombocytopenia (5,000/mm^3^), with normal liver and kidney function tests and a normal general blood picture. Vitals of the patient were stable. Common tropical infectious causes for severe thrombocytopenia, like peripheral smear for malaria, ELISA test for scrub typhus, and blood cultures for enteric fever, were negative and were ruled out. There was no ultrasonographic evidence of splenomegaly to rule out malaria, enteric fever, and haematological malignancy. Upper gastrointestinal endoscopy showed gastropathy in the form of haemorrhagic spots and oesophageal ulcerations; however, the rapid urease test was negative. Autoimmune and other viral causes for severe thrombocytopenia were ruled out. Bone marrow examination of the patient showed cellular marrow with megakaryocytes, so a diagnosis of ITP secondary to dengue fever was made, given the patient’s history of dengue two months back and IgG ELISA positivity. The patient was initiated on IV steroids in the form of dexamethasone 40 mg daily for four days. Bleeding manifestations stopped, and thrombocytopenia recovered (215,000/mm^3^). Table 1 below describes the key clinical, haematological, and treatment response profiles of all four patients comprehensively.

**Table 1:** Details of all the cases

	Case 1	Case 2	Case 3	Case 4
Age (in years)	20	29	50	44
Gender	Female/Primigravida	Female/Primigravida	Female	Female
Comorbidities	None	None	Hypertension	Breast cancer
Symptom at presentation	Pain abdomen (Active labour)	Fever, abdominal pain	Fever, rash and gum bleed	Gum bleed, black stools
Platelet count at presentation (thou/cumm)	8.4	10	12	5
Treatment given	IV steroids	IV rituximab	IV steroids	IV rituximab
Platelet count on recovery (thou/cumm)	122	97	129	80
Outcome	Discharged	Discharged	Discharged	Discharged

## Discussion

This case series highlights a methodical approach to patients with dengue fever who present with persistent thrombocytopenia, even after the natural course of the disease has occurred. These patients develop secondary ITP, whose diagnosis and timely treatment can prevent catastrophic consequences.

Thrombocytopenia is a well-known haematological entity in dengue patients. It generally resolves after the seroconversion phase of dengue infection is over. Various pathophysiological mechanisms have been postulated for the same, including bone marrow suppression leading to reduced proliferative capacity of the platelet precursors. There is also activation of the complement system, leading to increased destruction. Some studies have shown activation of the intrinsic pathway of apoptosis, leading to platelet destruction [5].

The association of AIHA and ITP was first described by Robert Evans in 1951 and is known as Evans syndrome [6]. Pathophysiology of this syndrome includes activation of autoreactive T lymphocytes and production of autoantibodies by the B lymphocytes [7].

The majority of cases of Evans syndrome are associated with autoimmune diseases such as common variable immunodeficiency, lymphoproliferative diseases, and systemic lupus erythematosus, with a higher prevalence in the paediatric population [7]. The first line of treatment for Evans Syndrome includes oral corticosteroids at a dose of 1-2 mg/kg/day and IV immunoglobulin at a dose of 2 g/kg over five days. The second line of management includes other immunosuppressive therapies along with rituximab [8]. The association between haemolytic anemia and ITP in dengue fever is a rare entity with very few cases reported [9,10].

ITP is a diagnosis of exclusion for thrombocytopenia when various causes have been ruled out. Dengue fever has been reported in patients with ITP [11]. Numerous causes for secondary ITP have been postulated, and one of them has been reported as dengue fever [12]. ITP, being a diagnosis of exclusion, is either incidentally diagnosed or concluded after a thorough workup of patients presenting with profuse bleeding manifestations.

## Conclusions

Persistent thrombocytopenia during the dengue season is usually caused by bone marrow suppression secondary to dengue fever, leading to a delay in diagnosing alternative etiologies such as ITP. Our case series highlights the importance of a rigorous evaluation of patients who have thrombocytopenia, even when they have recovered from the illness, underlining the importance of missing an alternative diagnosis, resulting in prolonged morbidity and incorrect management.

It is crucial for clinicians to think of a broad differential diagnosis in patients with dengue fever, especially when thrombocytopenia persists after recovery from the febrile phase, and the clinical course, laboratory findings, and peripheral blood smear do not line up with the typical course of dengue fever. Diagnosing and treating ITP or Evans syndrome with early initiation of steroid therapy prevents impending complications and improves clinical outcomes. A widespread awareness regarding these possible causes is essential to avoid impediments in the earlier diagnosis and recovery of patients.
